# Draft genome of six tropical *Azospirillum argentinense* strains associated with maize and sorghum with potential for plant growth promotion

**DOI:** 10.1128/mra.00332-26

**Published:** 2026-05-29

**Authors:** Valter Cruz-Magalhães, Vitória Palhares Ribeiro, Ubiraci Gomes de Paula Lana, Lourenço Vitor Silva Ferreira, Mikaely Sousa Marins, Maycon Campos Oliveira, José Edson Fontes Figueiredo, Denise Pacheco dos Reis, Gisele de Fátima Dias Diniz, Caroline dos Santos Martins Guieiro, Francisco Adriano de Souza, Eliane Aparecida Gomes, Christiane Abreu de Oliveira Paiva, Sylvia Morais de Sousa, Ivanildo Evódio Marriel

**Affiliations:** 1 Embrapa Maize and Sorghum 592341 , Sete Lagoas, Minas Gerais, Brazil; 2 Federal University of São João del-Rei 74383, São João del Rei, Minas Gerais, Brazil; 3 Federal University of Lavras (UFLA) 67739, Lavras, Minas Gerais, Brazil; Queens College Department of Biology, Queens, New York, USA

**Keywords:** biostimulants, biological nitrogen fixation, diazotrophs, plant growth-promoting bacteria

## Abstract

This report presents draft genome sequences of six *Azospirillum argentinense* strains (CMS1626, CMS1630, CMS05, CMS07, CMS11, and CMS18) isolated from maize rhizosphere soil or from sorghum stems grown on oxisols in the Brazilian Cerrado. These diazotrophic strains are promising bioinoculants for sustainable agriculture.

## ANNOUNCEMENT

Bioinoculants based on plant growth-promoting bacteria are increasingly used to support productive and sustainable agriculture (1, 2). Among them, *Azospirillum* is widely recognized for improving crop productivity through biological nitrogen fixation, enhanced nutrient availability, stress tolerance, and plant growth promotion (3, 4). *Azospirillum* spp. can fix atmospheric nitrogen, reducing dependence on chemical fertilizers (5), and produce phytohormones such as indole-3-acetic acid, which stimulate root development and nutrient uptake (6–8), enhancing plant resilience under suboptimal conditions (8, 9).

Here, we report the draft genome sequences of six *Azospirillum argentinense* strains isolated from maize rhizosphere soil and sorghum stems (Table 1). Samples were collected at the flowering stage from field experiments conducted at the Embrapa Maize and Sorghum Experimental Station in Sete Lagoas, Minas Gerais, Brazil (19°28′ S, 44°15′ W; 761 m altitude). The soil is classified as a typical dystrophic Red Oxisol (Soil Taxonomy), Cerrado phase (10).

**TABLE 1:** List of bacterial strains, summary of genome features, accession numbers, and general statistics of raw sequences and assembled genomes of *Azospirillum argentinense* strains CMS1626, CMS1630, CMS05, CMS07, CMS11, and CMS18

Strain ID	CMS1626	CMS1630	CMS05	CMS07	CMS11	CMS18
Best match (type species)	*A. argentinense*	*A. argentinense*	*A. argentinense*	*A. argentinense*	*A. argentinense*	*A. argentinense*
Fast ANI placement (%)	98.54	98.62	98.54	98.56	98.57	98.92
Fast ANI reference (NCBI)	Az39	Az39	Az39	Az39	Az39	Az39
Library size	13,640,092	13,656,034	13,649,444	13,686,604	13,698,520	13,666,770
Number of contigs	164	193	155	167	153	159
Genome size (bp)	7,755,591	7,331,621	7,474,862	7,476,534	7,413,709	7472015
Total sequence length (bp)	13,640,092	13,656,034	13,649,444	13,686,604	13,698,520	13666770
Total ungapped length (bp)	7,726,501	7,331,521	7,474,666	7,457,545	7,384,753	7452289
N50 (kb)	271.131	302.722	403.991	428.079	496.634	271.160
G + C content (%)	68.43	68.64	68.54	68.54	68.68	68.66
Genes (CDS)	6954	6497	6602	6602	6564	6578
Non-coding (RNA)	97	104	104	103	99	104
Genome completeness (%)	99.2%	99.2%	99.2%	99.2%	99.2%	99.2%
Genome contamination (%)	3.29	3.07	4.61	4.61	3.73	3.51
Coverage (×)	248	265	254	259	263	256
SRA identifiers	SRS24755893	SRS27295600	SRS24719299	SRS24752666	SRS24761364	SRS24755893
Genome assembly (NCBI)	GCA_054048325.1	GCA_054048285.1	GCA_054048425.1	GCA_054065635.1	GCA_054504525.1	GCA_054048405.1
BioSample ID	SAMN47891599	SAMN47891619	SAMN47891644	SAMN47891646	SAMN44516662	SAMN47891450
GenBank accession number	JBSVFC010000001.1	JBSVFE010000001.1	JBSVFF010000001.1	JBSVFB010000001.1	JBIUFN010000001.1	JBSVFD010000001.1
Strain	BRM 044311	BRM 044312	BRM 044317	BRM 044318	BRM 044319	BRM 044320
Isolate name alias	CMPC 1821	CMPC 1822	CMPC 1827	CMPC 1828	CMPC 1829	CMPC 1830
Country/state/municipality	Brazil/MG/Sete Lagoas	Brazil/MG/Sete Lagoas	Brazil/MG/Sete Lagoas	Brazil/MG/Sete Lagoas	Brazil/MG/Sete Lagoas	Brazil/MG/Sete Lagoas
Geographic location	19° 28′S, 44° 15′W	19° 28′S, 44° 15′W	19° 28′S, 44° 15′W	19° 28′S, 44° 15′W	19° 28′S, 44° 15′W	19° 28′S, 44° 15′W
Biome	Cerrado	Cerrado	Cerrado	Cerrado	Cerrado	Cerrado
Collection date	2011	2012	1996	1996	1996	1996
Host	Sorghum stem	Sorghum stem	Maize rhizosphere soil	Maize rhizosphere soil	Maize rhizosphere soil	Maize rhizosphere soil
Isolation medium	NFb	NFb	NFb	NFb	NFb	NFb

Sorghum stems were harvested, washed, surface-disinfected, and aseptically macerated. Maize plants were collected with roots and adhering soil intact, and the rhizosphere soil was separated (11). For bacterial isolation, 10 g of each sample was suspended in 90 mL sterile saline solution (8.5 g L⁻¹ NaCl), shaken at 150 rpm for 30 min, and allowed to settle for 1 h. Serial dilutions were prepared up to 10⁻⁷.

Aliquots (100 µL) were inoculated into vials containing 5 mL of semi-solid nitrogen-free NFb medium, as described by Baldani and Döbereiner (12), and incubated at 30°C for 5 days. Diazotrophic growth was identified by the formation of a subsurface pellicle. Cultures were purified through approximately five successive transfers in NFb medium, and purity was confirmed on solid potato medium (12).

Strains were cryopreserved at −80°C in glycerol and deposited in the Embrapa Collection of Multifunctional and Phytopathogenic Microorganisms under accession numbers BRM 044320 (CMS18), BRM 044317 (CMS05), BRM 044312 (CMS1630), BRM 044311 (CMS1626), BRM 044318 (CMS07), and BRM 044319 (CMS11).

Genomic DNA was extracted from cultures grown in Luria-Bertani medium (28°C, 24 h, 150 rpm) using the Wizard Genomic DNA Purification Kit (Promega, USA) and quantified with a Qubit 2.0 fluorometer (Life Technologies, USA). Whole-genome sequencing libraries were prepared following the BGISEQ-500 protocol described by Huang et al. (13), including DNA fragmentation and size selection (200–400 bp) using Agencourt AMPure XP magnetic beads (Beckman Coulter, USA). Libraries were sequenced on a BGISEQ-500 platform (150-bp paired-end) at BGI (Shenzhen, China), and base calling was performed using the platform’s internal software.

Low-quality reads (Phred score <20) were removed using Trimmomatic v0.38 (14). Genome assembly was performed with SPAdes v3.12.0 (15), and assembly quality and completeness were assessed using Quast v5.0.2 (16) and BUSCO v5.3.1 (17), respectively. Genomes were annotated with the National Center for Biotechnology Information (NCBI) Prokaryotic Genome Annotation Pipeline v6.6 (18). Species identification was conducted using TYGS v39.1 (19) against type strains in the RefSeq database (October 2024) and confirmed using fastANI v1.34 (20) against *A. argentinense* Az39 (NZ_CP007793.1). General genome features and ANI results are summarized in Table 1. All software was used with default parameters.

## Data Availability

This Whole Genome Shotgun project has been deposited at DDBJ/ENA/GenBank under BioProject PRJNA1180210, with accession JBIUFN000000000, and BioSample accessions SAMN47891599, SAMN47891619, SAMN47891644, SAMN47891646, SAMN44516662, and SAMN47891450 for strains CMS1626, CMS1630, CMS05, CMS07, CMS11, and CMS18, respectively. The raw reads were deposited in the SRA database and can be accessed under the numbers SRS24755893 (CMS1626), SRS27295600 (CMS1630), SRS24719299 (CMS05), SRS24752666 (CMS07), SRS24761364 (CMS11), and SRS24755893 (CMS18).

## References

[1] ALKahtani MDF, Fouda A, Attia KA, Al-Otaibi F, Eid AM, Ewais E-D, Hijri M, St-Arnaud M, Hassan S-D, Khan N, Hafez YM, Abdelaal KAA. 2020. Isolation and characterization of plant growth promoting endophytic bacteria from desert plants and their application as bioinoculants for sustainable agriculture. Agronomy 10:1325. doi:10.3390/agronomy10091325

[2] Agbodjato NA, Babalola OO. 2024. Promoting sustainable agriculture by exploiting plant growth-promoting rhizobacteria (PGPR) to improve maize and cowpea crops. PeerJ 12:e16836. doi:10.7717/peerj.16836 38638155 PMC11025545

[3] Fukami J, Cerezini P, Hungria M. 2018. Azospirillum: benefits that go far beyond biological nitrogen fixation. AMB Express 8:73. doi:10.1186/s13568-018-0608-1 29728787 PMC5935603

[4] Pereg L, de-Bashan LE, Bashan Y. 2016. Assessment of affinity and specificity of Azospirillum for plants. Plant Soil 399:389–414. doi:10.1007/s11104-015-2778-9

[5] Cassán F, Coniglio A, López G, Molina R, Nievas S, de Carlan CLN, Donadio F, Torres D, Rosas S, Pedrosa FO, de Souza E, Zorita MD, de-Bashan L, Mora V. 2020. Everything you must know about Azospirillum and its impact on agriculture and beyond. Biol Fertil Soils 56:461–479. doi:10.1007/s00374-020-01463-y

[6] Pham T-M, Bui XD, Trang LVK, Le T-M, Nguyen ML, Trinh D-M, Phuong NTD, Khoo KS, Chew KW, Show PL. 2022. Isolation of indole-3-acetic acid-producing Azospirillum brasilense from Vietnamese wet rice: co-immobilization of isolate and microalgae as a sustainable biorefinery. J Biotechnol 349:12–20. doi:10.1016/j.jbiotec.2022.03.007 35331729

[7] Zaheer MS, Ali HH, Iqbal MA, Erinle KO, Javed T, Iqbal J, Hashmi MIU, Mumtaz MZ, Salama EAA, Kalaji HM, Wróbel J, Dessoky ES. 2022. Cytokinin production by Azospirillum brasilense contributes to increase in growth, yield, antioxidant, and physiological systems of wheat (Triticum aestivum l.). Front Microbiol 13:886041. doi:10.3389/fmicb.2022.886041 35663903 PMC9161363

[8] Mora V, López G, Molina R, Coniglio A, Nievas S, De Diego N, Zeljković SĆ, Sarmiento SS, Spíchal L, Robertson S, Wilkins O, Elías J, Pedraza R, Estevez JM, Belmonte MF, Cassán F. 2023. Azospirillum argentinense modifies Arabidopsis root architecture through auxin-dependent pathway and flagellin. J Soil Sci Plant Nutr 23:4543–4557. doi:10.1007/s42729-023-01371-8

[9] Méndez-Gómez M, Barrera-Ortiz S, Castro-Mercado E, López-Bucio J, García-Pineda E. 2021. The nature of the interaction Azospirillum-Arabidopsis determine the molecular and morphological changes in root and plant growth promotion. Protoplasma 258:179–189. doi:10.1007/s00709-020-01552-7 33009649

[10] Santos HG, Jacomine PKT, Anjos LHC, Oliveira VA, Lumbreras JF, Coelho MR, Almeida JA, Cunha TJF, Oliveira JB. 2013. Sistema brasileiro de classificação de solos. Brasília Embrapa

[11] Ribeiro VP, Gomes EA, de Sousa SM, de Paula Lana UG, Coelho AM, Marriel IE, de Oliveira-Paiva CA. 2022. Co-inoculation with tropical strains of Azospirillum and Bacillus is more efficient than single inoculation for improving plant growth and nutrient uptake in maize. Arch Microbiol 204:143. doi:10.1007/s00203-022-02759-3 35044594

[12] Baldani VLD, Döbereiner J. 1980. Host-plant specificity in the infection of cereals with Azospirillum spp. Soil Biol Biochem 12:433–439. doi:10.1016/0038-0717(80)90021-8

[13] Huang J, Liang X, Xuan Y, Geng C, Li Y, Lu H, Qu S, Mei X, Chen H, Yu T, Sun N, Rao J, Wang J, Zhang W, Chen Y, Liao S, Jiang H, Liu X, Yang Z, Mu F, Gao S. 2020. BGISEQ-500 （DNBSEQ-G50）WGS library construction v1. Protocols.io. doi:10.17504/protocols.io.bim3kc8n

[14] Bolger AM, Lohse M, Usadel B. 2014. Trimmomatic: a flexible trimmer for Illumina sequence data. Bioinformatics 30:2114–2120. doi:10.1093/bioinformatics/btu170 24695404 PMC4103590

[15] Prjibelski A, Antipov D, Meleshko D, Lapidus A, Korobeynikov A. 2020. Using SPAdes de novo assembler. Curr Protoc Bioinformatics 70:e102. doi:10.1002/cpbi.102 32559359

[16] Mikheenko A, Prjibelski A, Saveliev V, Antipov D, Gurevich A. 2018. Versatile genome assembly evaluation with QUAST-LG. Bioinformatics 34:i142–i150. doi:10.1093/bioinformatics/bty266 29949969 PMC6022658

[17] Manni M, Berkeley MR, Seppey M, Zdobnov EM. 2021. BUSCO: assessing genomic data quality and beyond. Curr Protoc 1:e323. doi:10.1002/cpz1.323 34936221

[18] Tatusova T, DiCuccio M, Badretdin A, Chetvernin V, Nawrocki EP, Zaslavsky L, Lomsadze A, Pruitt KD, Borodovsky M, Ostell J. 2016. NCBI prokaryotic genome annotation pipeline. Nucleic Acids Res 44:6614–6624. doi:10.1093/nar/gkw569 27342282 PMC5001611

[19] Meier-Kolthoff JP, Göker M. 2019. TYGS is an automated high-throughput platform for state-of-the-art genome-based taxonomy. Nat Commun 10:2182. doi:10.1038/s41467-019-10210-3 31097708 PMC6522516

[20] Jain C, Rodriguez-R LM, Phillippy AM, Konstantinidis KT, Aluru S. 2018. High throughput ANI analysis of 90K prokaryotic genomes reveals clear species boundaries. Nat Commun 9:5114. doi:10.1038/s41467-018-07641-9 30504855 PMC6269478

